# BEHAVIOR CHANGE TECHNIQUES IN PERSONS WITH MCI AND MD AND THEIR CARE PARTNERS: A REPORT OF AN ORAL HEALTH INTERVENTION

**DOI:** 10.1093/geroni/igad104.1324

**Published:** 2023-12-21

**Authors:** Ashley Bryant, Youngmin Cho, courtney caiola, Donald Bailey, Brenda Plassman, Bei Wu, Ruth Anderson

**Affiliations:** The University of North Carolina at Chapel Hill, Chapel Hill, North Carolina, United States; University of North Carolina at Chapel Hill, Chapel Hill, North Carolina, United States; East Carolina University, Greenville, North Carolina, United States; Duke University, Durham, North Carolina, United States; Duke University, Durham, North Carolina, United States; New York University, New York City, New York, United States; University of North Carolina at Chapel Hill, Chapel Hill, North Carolina, United States

## Abstract

Older adults with cognitive impairment are likely to experience a decline in oral health and hygiene, which can have profound negative impacts such as cavities, gum disease and tooth loss. Poor oral health is also linked to adverse health outcomes including diabetes, cardiovascular disease, and aspiration pneumonia. Despite these factors, inadequate oral hygiene practices provided by formal and informal care partners may contribute to poor oral hygiene. In this study, we aimed to improve oral hygiene outcomes (plaque index and gingival index) for individuals with mild cognitive impairment (MCI) or mild dementia (MD) by fostering behavior changes among care partners trained to assist them. We identified and compared active behavior change techniques (BCT) used in a care partner-assisted oral health intervention for individuals with MCI and MD using a qualitative, secondary analysis of coaching sessions with care partners (n=17 dyads: 10 dyads for MCI, 7 dyads for MD). Frequently used BCTs in both MCI and MD groups were prompts and cues, instruction on how to perform the behavior, review behavioral goal, and problem solving. Common BCTs were found across both MCI and MD groups that provide clarity on the active components for behavior change. Social support-unspecified emerged for MCI group and credible source emerged for MD group. In this intervention, BCTs were focused on oral health, however BCTs can assist in addressing multiple types of behavior change. Findings from this study provide insight into the mechanisms of changes in individuals’ behaviors using these interventions.

